# Epidemic Conjunctivitis

**Published:** 1880-08

**Authors:** Lester Curtis

**Affiliations:** No. 1558 Wabash Avenue, Chicago


CHICAGO, July, 1880.

---

### ARTICLE VII.

*Epidemic Conjunctivitis.*

I have lately met with a number of cases of inflammation of the conjunctiva, which, in some respects, differ from ordinary conjunctivitis.

These cases seem to be the results of some epidemic influence. Persons are attacked without having in any way changed their usual habits of life or been exposed to cold, dust, contagion or any other discoverable cause.

The onset of the disease is tolerably rapid. The first symptom is a slight feeling of irritation at the inner canthus of one eye, with a slight injection of the vessels. The injection increases rapidly, and, in from six to eight hours spreads over the whole conjunctiva, the redness does not, however, become so intense as to prevent the white sclerotic from shining through. The feeling of irritation does not increase with the redness, it rather grows less, and there is never found during the whole course of the disease the feeling of sand or sticks in the eye, so characteristic of ordinary conjunctivitis.

Very soon after the first sign of redness of the conjunctiva, a creamy, yellowish-white discharge appears. At first this is scanty and lies in little rolls in the palpebral folds. These rolls are forced out by the movements of the lid and cause considerable annoyance by coming in front of the pupil and obstructing the field of view, sometimes the discharge is spread in a thin layer over the front of the cornea. This can easily be removed and then the vision appears to be as good as ever until it re-appears. Seen under the microscope the discharge shows all the characteristic of ordinary pus. Soon after the beginning of the discharge the lids begin to swell, sometimes swelling enough to close the eye.

At first there is no apparent interference with vision except the mechanical ones first mentioned, but after a few hours a mist before the eye begins to be noticed after looking intently at any object for a short time. This fog can be dissipated by an effort or by the appearance of any object which strongly fixes the attention, but reappears much more dense in a short time, or when the attention is relaxed. After resting the vision becomes clear again.

If the attempt is made to continue the use of the eyes after the appearance of the fog, a feeling of fatigue is experienced which soon increases to one of actual pain. After a few hours, vision becomes so indistinct that it is difficult to see minute objects clearly and larger ones appear to be surrounded by a halo and more or less of a fog envelops every thing. By a strong effort of attention this fog can be partly dissipated but not entirely.

In from two to three days the swelling of the lids disappears, the discharge and injection of the conjunctiva remain for a week or so longer. The difficulty of using the eyes, however, remains for a considerable time after all other symptoms have dissappeared.

In some cases the swelling of the lids and the discharge is less but there are paroxysms of pain in the eye. These paroxysms have a certain periodical character, the patient being free from them for a considerable portion of the day, but feeling them come on at some pretty definite time, usually late in the afternoon. Some of these cases are troubled with double vision, or rather multiple vision, seeing many objects, half a dozen in some cases in place of one. Occasionally there is a sort of photophobia. Such patients prefer to be in a darkened room, a slight increase in the intensity of the light causes a sensation of not exactly pain but rather of dazzling, as from a very bright light.

I have seen cases in which the trouble was confined to one eye, especially in the lighter cases; but usually in a few hours after the beginning of the trouble in one eye, it appears in the other also, running the same course though often somewhat lighter.

The cases seem to be accompanied with no special derangement of health, so far as I can discover.

I have heard of these cases in almost every quarter of the city. And lately, on a visit to central New York, I learned that there had been a number of similar cases there.

The treatment that has seemed to succeed best has been the instillation of a solution of borax in rose-water or in ordinary distilled water, of the strength of ten grains to the ounce, every two or three hours. The addition of a little atropia seems to relieve the cases attended with pain. LESTER CURTIS.

NO. 1558 WABASH AVENUE, CHICAGO, July, 1880.

---

ESSENCE OF WINTERGREEN AS AN ANTISEPTIC.—M. Perier. (*Journ. de Méd.*, May, 1880.)—The essence of wintergreen, on account of its powerful antiseptic action and its agreeable odor, is recommended by M. Perier. He uses it mixed with vaseline, 1 part to 100, for lubricating instruments, and his hands for vaginal examinations, and prefers it to carbolic acid. He also uses a solution of it for bathing wounds and for simple dressings.

REMEDY FOR ACUTE CORYZA.—M. Yron. *Journ de Méd.*, May, 1880.—The author recommends the following powder to be used as snuff:

| | | |
|---|---|---|
| Subnitrate of bismuth | 20 | grams. |
| Powdered benzoin | 10 | " |
| Tannin | 4 | " |
| Chlorhydrate of morphine | 0 | 10 centigrams. |

